# Roles of Extracellular Vesicle-Derived microRNAs in Metabolic Dysfunction-Associated Steatotic Liver Disease to Hepatocellular Carcinoma

**DOI:** 10.3390/biomedicines14030528

**Published:** 2026-02-26

**Authors:** Xinlei Ma, Wei Zheng, Chensi Wu, Chengan Xu, Xin Ji, Keyang Xu, Qiaoqiao Yin

**Affiliations:** 1 Department of Infectious Diseases, Zhejiang Provincial People’s Hospital, Hangzhou 310014, China; xinleima@zju.edu.cn (X.M.);; 2 State Key Laboratory of Quality Research in Chinese Medicine, Faculty of Chinese Medicine, Macau University of Science and Technology, Macau 999078, China

**Keywords:** extracellular vesicle, microRNAs, metabolic dysfunction-associated steatotic liver disease, hepatocellular carcinoma

## Abstract

Metabolic dysfunction-associated steatotic liver disease (MASLD) is a leading cause of chronic liver disease, and has emerged as a common etiological factor for hepatocellular carcinoma (HCC). MASLD and MASLD-associated HCC lack specific clinical biomarkers. Extracellular vesicles (EVs) and their microRNA (miRNA) cargo have emerged as key mediators of intercellular communication and promising diagnostic tools. This review provides a systematic overview of the stage-specific roles of EV-derived miRNAs across the MASLD spectrum. We focus on how key EV-miRNAs regulate lipid metabolism, inflammatory responses, hepatic stellate cell (HSC) activation, and the remodeling of the tumor microenvironment (TME). This review provides an updated perspective on cross-stage EV-derived miRNA regulatory circuits. In addition, we critically evaluate the potential of EV-derived miRNAs as non-invasive biomarkers and therapeutic targets. By integrating mechanistic insights with clinical relevance, this review provides a comprehensive framework for the early identification, risk stratification, and precision intervention of MASLD-associated HCC.

## 1. Introduction

Metabolic dysfunction-associated steatotic liver disease (MASLD), formerly known as nonalcoholic fatty liver disease (NAFLD), represents a chronic, metabolism-driven liver injury that develops in genetically susceptible individuals as a consequence of long-standing metabolic stress and insulin resistance. The disease spectrum encompasses metabolic dysfunction-associated steatotic liver (MASL), metabolic dysfunction-associated steatohepatitis (MASH), and associated cirrhosis and hepatocellular carcinoma (HCC). MASLD has become the leading cause of chronic liver disease globally due to societal development and lifestyle changes, including obesity, insulin resistance, and sedentary behavior. Epidemiologically, the global prevalence of MASLD increased significantly from 25.26% in 1990–2006 to 38.2% in 2016–2019, with an exponential increase in economic burden [1,2]. MASLD is characterized by hepatic steatosis with at least one of five cardiometabolic risk factors, such as obesity, lipidemia, hypertension, insulin resistance, and type 2 diabetes. Cardiovascular disease (CVD), cancer, and liver failure are the leading causes of death in MASLD [3,4]. As the sixth most common cancer globally, the incidence of HCC is continuing to rise steadily. With the widespread clinical success of anti-hepatitis B virus (anti-HBV) therapy, MASLD is rapidly becoming a common cause of chronic liver disease, cirrhosis, and HCC [5,6]. A subset of MASLD patients experience inflammation, fibrosis, and even cirrhosis, which can eventually progress to HCC. However, certain patients may also progress to HCC without cirrhosis [7]. MASLD often presents with no specific clinical signs. Evaluation of liver fibrosis and steatosis is crucial for the diagnosis and staging of MASLD. However, unlike viral liver disease, there are currently no specific markers for MASLD. Therefore, the effective management of MASLD remains a significant challenge.

It should be noted that, with a deeper understanding of the metabolic and pathophysiological basis of this disease spectrum, international organizations have in recent years proposed and promoted a revised nomenclature centered on MASLD, which more accurately reflects the metabolic drivers of the disease. Terminology carrying stigmatizing implications, such as NAFLD and metabolic dysfunction-associated fatty liver disease (MAFLD), is therefore being used less frequently. However, given the wide time span of published studies, earlier terms, including NAFLD, non-alcoholic steatohepatitis (NASH), simple steatosis (SS), and isolated liver steatosis (ILS), remain prevalent in the existing literature. To ensure consistency and minimize conceptual confusion, MASLD is used as the primary term throughout this review, whereas legacy terminology is retained only when citing original studies or specific historical contexts.

As carriers of intercellular information transmission, extracellular vesicles (EVs) can transport a variety of substances, including proteins, mRNA, non-coding RNAs (ncRNAs), DNA, and lipids [8]. Recent studies indicate that EVs play an important role in the occurrence and development of chronic liver diseases, including MASLD, alcoholic liver disease, liver fibrosis, and HCC [9]. EV-enclosed ncRNAs exhibit high stability and a strong correlation with chronic liver disease. As the main component of small ncRNAs, microRNA (miRNA) has been widely recognized for its regulation of gene expression [10].

This narrative review is based on a comprehensive survey of recently published literature from major biomedical databases, including PubMed and Web of Science, and integrates representative findings from both basic and clinical studies. The literature search employed combinations of the following keywords: “MASLD”, “MASH”, “NAFLD”, “NASH”, “steatosis”, “fibrosis”, “cirrhosis”, and “HCC” together with “extracellular vesicles”, “exosomes”, “microvesicles”, and “microRNA”. Reference lists of relevant articles were also manually screened to identify additional pertinent studies.

This review focuses on the stage-specific alterations of EV-derived miRNAs across the spectrum of MASLD and on the molecular networks through which they link metabolic dysregulation, chronic inflammation, fibrotic remodeling, and hepatocarcinogenesis.

Priority was given to studies that explicitly characterized EV-associated or exosome-enriched miRNAs, including those with defined EV isolation methods or vesicle-marker validation. When EV-specific evidence was limited, selected studies on hepatic or circulating miRNAs were included to provide a biological context. This review highlights that EV-derived miRNAs are not merely passive indicators of disease state but also active regulators driving pathological progression. Investigating EV-derived miRNAs as non-invasive diagnostic tools, risk-stratification markers, and therapeutic targets offers novel avenues for precision medicine in MASLD and MASLD-associated HCC.

## 2. Pathogenesis of MASLD-Associated HCC

With the emphasis on viral hepatitis management and the rising prevalence of metabolic diseases, the incidence of MASLD-associated HCC is escalating globally [11,12]. MASLD is not a discrete pathological entity but rather a clinical spectrum, ranging from MASL and MASH to fibrosis to cirrhosis, eventually predisposing to HCC. A distinctive feature of MASLD-associated HCC is that approximately one-third of cases develop in the absence of underlying liver cirrhosis [7]. During disease progression, interactions among different cell types in the liver play a critical role. Hepatocytes, the primary parenchymal cells, account for 70–80% of liver mass and are central to lipid and glucose homeostasis. Non-parenchymal cells, including Kupffer cells (resident macrophages), hepatic stellate cells (HSCs), liver sinusoidal endothelial cells (LSECs), and cholangiocytes, support immune regulation, fibrogenesis, vascular integrity, and bile secretion, respectively. In MASLD progression, hepatocytes undergo lipid accumulation and subsequent lipotoxic injury. Kupffer cells and HSCs are activated, promoting inflammation and fibrosis through cytokine release and extracellular matrix (ECM) deposition. LSECs lose their fenestrations and form a basement membrane, impairing nutrient and oxygen exchange. Cholangiocytes expand and remodel, further contributing to the pro-fibrotic environment.

Chronic inflammation is thought to be a primary driver of the progression from MASL/MASH to HCC, leading to persistent liver damage, subsequent fibrosis, and malignant transformation. The key mechanisms involved include:

(i) Lipid accumulation-induced chronic inflammation: Due to metabolic imbalance, lipids accumulate in hepatocytes, triggering a stress response of hepatocytes, which leads to the recruitment of resident immune cells in the liver. The release of pro-inflammatory cytokines (such as tumor necrosis factor-α (TNF-α) and Interleukin-6 (IL-6) triggers responses which, in turn, attract additional immune cells and form a cycle of chronic inflammation that converts metabolic stress into sustained immune activation [13].

(ii) Oxidative stress and DNA damage: Inflammation leads to a large amount of oxidative stress, primarily via the production of reactive oxygen species (ROS) and reactive nitrogen species (RNS) [14]. This results in lipid peroxidation, mitochondrial dysfunction, DNA mutations, and genome instability [15]. Such DNA damage not only accelerates hepatocyte death but also promotes the clonal expansion of cells with oncogenic potential, highlighting oxidative stress as a central driver of MASLD-HCC progression.

(iii) Activation of immune cells: The immune system is the core of maintaining inflammation and HCC transformation, particularly via macrophages and T cells [16], which can damage the liver after entering liver tissue. These cells infiltrate the hepatic parenchyma, amplifying local immune responses and creating a pro-tumorigenic microenvironment.

(iv) Hepatic fibrosis: One of the most critical factors in MASLD-HCC transformation during fibrosis. Persistent inflammation activates the transformation of HSCs into myofibroblasts, leading to ECM deposition and fibrosis, and some untreated cases may lead to cirrhosis [17]. Fibrosis not only disrupts liver architecture but also creates a stiff, pro-tumorigenic matrix that facilitates cancer cell invasion and survival.

(v) Metabolic dysfunction-related inflammation and carcinogenesis: The mitogen-activated protein kinase (MAPK) and phosphoinositide 3-kinase (PI3K)-Protein kinase B (Akt)-mammalian target of rapamycin (mTOR) pathways are upregulated in MASLD [18], promoting the survival and proliferation of abnormal cells. Furthermore, novel regulators, such as the tumor necrosis factor receptor-associated factor 6 (TRAF6)-binding protein (T6BP), have been shown to suppress MASLD-HCC progression [19]. Patients with MASLD showed higher hepatic expression of the protein tyrosine phosphatase receptor type K (PTPRK), while *Ptprk*-knockout mice on a high-fat diet developed smaller tumors during hepatocarcinogenesis [20]. T6BP and PTPRK are emerging pathogeneses of MASLD-associated HCC and may serve as future therapeutic targets.

(vi) Liver microenvironment and pro-carcinogenic signaling pathways: Persistent inflammation renders the liver microenvironment rich in cytokines and growth factors, thereby activating pathways such as Janus kinase/signal transducer and activator of transcription (JAK/STAT) and nuclear factor kappa-light-chain-enhancer of activated B cells (NF-κB). These signals promote hepatocyte proliferation and survival, while attenuating apoptosis [21].

These collective pathological alterations provide favorable conditions for the progression of MASLD to HCC.

## 3. EVs

EVs are membrane-bound structures released by cells into the extracellular space. Their size, molecular composition, and surface characteristics are strongly influenced by the cell of origin and its physiological or pathological state. Traditionally, EVs have been categorized as exosomes or microvesicles based on their presumed biogenesis and size range. However, growing evidence indicates that such subtype classifications are mainly operational, as different subtypes overlap extensively in size distribution, molecular markers, and isolation methods, making a clear distinction difficult under experimental conditions. Therefore, current research tends to view EVs as a continuous functional population, with their biological effects determined primarily by the cell of origin and the bioactive molecules they carry, rather than by their presumed biogenetic subtype [8,22]. In line with current consensus recommendations, the term “EVs” is used throughout this manuscript unless otherwise specified. EVs carry a broad range of bioactive molecules, including proteins, lipids, DNA, messenger RNAs, and ncRNAs. Through cargo transfer, EVs enable efficient communication between neighboring cells and distant organs [23].

EV-mediated signaling plays an important role in the regulation of physiological homeostasis and disease progression. EVs participate in metabolic regulation, immune responses, inflammation, and tissue remodeling by delivering functional molecules to recipient cells [24,25,26]. In metabolic disorders, EVs derived from adipose tissue act as endocrine-like mediators and transmit signals to peripheral organs. Notably, adipose tissue-derived EVs have been shown to communicate with the brain and promote neuroinflammation and cognitive impairment associated with insulin resistance, highlighting the systemic effects of EV-mediated inter-organ communication [27]. In cancer, EVs are increasingly recognized as active contributors to tumor progression rather than passive byproducts of malignant cells. Tumor-derived EVs modulate the tumor microenvironment by promoting angiogenesis, reprogramming stromal and immune cells, and facilitating immune evasion. In addition, EVs support tumor dissemination by preparing pre-metastatic niches and enhancing metastatic potential. These findings underscore the central role of EVs in coordinating local and systemic tumor-promoting signals during cancer development [28,29].

Beyond their biological functions, EVs have attracted significant attention for their clinical relevance. EVs are stable in body fluids and protect their cargo from enzymatic degradation, making them well-suited for minimally invasive liquid biopsy approaches. Importantly, EV cargo profiles often reflect disease-specific molecular changes in the originating cells. Among these cargos, EV-encapsulated miRNAs are of particular interest because of their high stability and reproducibility in circulation. Accumulating evidence supports the potential of EV-derived miRNAs as biomarkers for disease diagnosis, prognosis evaluation, and therapeutic response monitoring [22,30]. Moreover, the intrinsic biocompatibility and low immunogenicity of EVs make them attractive candidates for drug delivery and therapeutic applications.

EVs constitute a versatile and efficient communication system that integrates metabolic, inflammatory, and oncogenic signals across tissues.

## 4. MiRNAs

Among the diverse cargos carried by EVs, miRNAs have emerged as pivotal functional mediators of EV-driven intercellular communication [31].

MiRNAs are small nc RNAs transcribed from intergenic or intragenic genomic regions. Their biogenesis begins in the nucleus, where primary miRNAs (pri-miRNAs) are processed by the RNase III endonuclease Drosha into precursor miRNAs (pre-miRNAs). Following nuclear export, pre-miRNAs are further cleaved by Dicer in the cytoplasm to generate mature miRNAs, which are subsequently incorporated into Argonaute-containing RNA-induced silencing complexes (RISCs). By inducing translational repression or mRNA degradation, miRNAs function as a central regulator of post-transcriptional gene expression, facilitating rapid cellular adaptations to physiological stress, metabolic perturbations, and inflammatory signaling [32].

Beyond their intracellular regulatory roles, miRNAs can also be released into the extracellular environment and persist in the circulation in several stabilized forms. Among these, miRNAs encapsulated within EVs are effectively protected from extracellular RNase-mediated degradation, maintaining their detectability and stability in blood and other body fluids [33,34]. Notably, while EV-derived miRNAs constitute an important subset of circulating miRNAs, they do not contribute their only extracellular form. Compared with circulating miRNAs complexed with RNA-binding proteins or lipoproteins, EV-derived miRNAs exhibit distinct advantages in terms of molecular stability, delivery efficiency, and potential biological potency. Accordingly, EV-derived miRNAs are widely regarded as a key molecular basis for EV-mediated long-range intercellular communication.

Under specific physiological or pathological conditions, cells can selectively package defined miRNA species into EVs and release them into the circulation, enabling their transfer to recipient cells across tissues. Through this mode of communication, EV-derived miRNAs contribute to the regulation of diverse biological processes, including cellular stress responses, signal transduction, metabolic homeostasis, and immune modulation [35]. Recent studies have further demonstrated that the loading of miRNAs into EVs is a tightly regulated and selective process rather than a random event. This selective sorting is influenced by miRNA sequence motifs, RNA-binding proteins, and mechanisms associated with EV biogenesis, resulting in disease-specific EV-derived miRNA profiles [36].

Due to their abundance, high stability, and accessibility, along with the close association with disease-related pathological molecular shifts, EV-derived miRNAs are considered ideal candidates for biomarker development [37]. With advances in experimental technologies, increasing evidence has clarified the functional roles of EV-derived miRNAs across the MASLD-to-HCC spectrum, supporting their relevance in both mechanistic studies and clinical translation.

## 5. EV-Derived miRNAs in MASL and MASH

One of the main features of MASL is dysregulated lipid metabolism, leading to excessive lipid accumulation in the liver. Lipid oxidation and oxidative phosphorylation generate excessive ROS, which activate inflammatory pathways, particularly the MAPK pathway, causing mitochondrial damage and hepatocyte injury. Mitochondrial dysfunction can further impair β-oxidation, resulting in lipotoxicity and cellular stress. In addition, abnormal mitochondria disrupt adenosine triphosphate (ATP) production, inhibit autophagy, and promote apoptosis, thereby exacerbating hepatocyte injury and fibrosis. As cellular damage accumulates and cytokines gather to amplify pro-inflammatory responses [38,39], driving MASL to MASH. Insulin resistance triggers and sustains hepatic lipid accumulation and inflammation, playing a central role in MASLD pathogenesis. Gastrointestinal hormones and intestinal flora are also thought to be related to MASLD [40].

The pathogenesis of early MASLD is multifactorial and is not fully understood. During lipid metabolism, free fatty acids (FFAs) are released to generate hepatotoxic reactive oxygen species, causing hepatocyte injury, which is a core feature of the pathogenesis of MASLD.

Type 2 diabetes is associated with peripheral insulin resistance, both of which are common in patients with MASLD, but not all patients with MASH have insulin resistance, suggesting that MASLD has a heterogeneous etiology.

Against the backdrop of lipotoxicity, insulin resistance, and progressive inflammation during the transition from MASL to MASH, increasing attention has been directed toward stage-specific changes in miRNA expression and function. These miRNAs participate in lipid metabolism, hepatocellular injury, and inflammatory signaling, thereby contributing to both disease initiation and progression.

Given that research specifically isolating EV-derived miRNAs is still emerging, comprehensively understanding their functions requires exploring their non-EV counterparts. Therefore, in the following sections, we explicitly delineate the biological context by discussing cellular, hepatic tissue-derived, and total circulating (or serum) miRNAs alongside EV-specific findings. This compartmentalized approach not only enriches the mechanistic landscape but also provides critical clues for deciphering the precise roles of EV-derived miRNAs in intercellular crosstalk. The following will highlight several representative EV-derived miRNAs and their molecular mechanisms

### 5.1. MiR-122

MiR-122 is the most abundant and highly tissue-specific miRNA in the liver. It is predominantly expressed in hepatocytes and accounts for approximately 60–70% of total miRNA content in normal liver tissue. Notably, miR-122 is also one of the most abundant and liver-enriched miRNAs packaged into EVs reported to date. During the MASL/MASH development, EV-derived miR-122 exhibits more dynamic changes with greater signaling significance compared to its intrahepatic expression. Importantly, serum miR-122 can arise not only from passive release following hepatocyte damage but also from active secretion into the bloodstream via EVs.

Compared with unfractionated serum miR-122, EV-derived miR-122 is more likely to represent an actively secreted and selectively packaged signaling form, reflecting and transmitting liver-derived metabolic and injury signals. Studies isolating liver-specific EVs show that the proportion of miR-122 in circulating EVs increases with disease progression, accounting for up to ~70% of hepatic miRNAs, suggesting that EV-derived miR-122 more accurately reflects liver-derived signals rather than passive release [41]. Further research demonstrates that miR-122 is significantly enriched in EVs from NAFLD patients, especially in adipocyte-derived exosomes (ADEs). Functional experiments show that ADEs-miR-122 promotes metabolic reprogramming by upregulating key lipogenic enzymes such as fatty acid synthase (FASN), glucose-6-phosphate dehydrogenase (G6PD), and ACC, and by directly targeting *SIRT1* 3′UTR to suppress its expression, thereby aggravating liver injury, inflammation, and fibrosis [42].

Moreover, miR-122 is mainly elevated in serum at early stages, while the difference between EVs and serum gradually diminishes as the disease progresses. In patients with high gamma-glutamyl transferase (GGT), EV-derived miR-122 levels increase, whereas decreased renal function (elevated creatinine) is associated with reduced EV-derived miR-122, suggesting that abnormal EV-mediated export of miR-122 may be linked to cholestasis and dysregulated systemic inflammation [43].

These findings suggest that EV-derived miR-122 not only serves as a passive marker of hepatocellular injury but also likely functions as a selectively packaged molecule involved in inter-tissue communication.

To fully appreciate the significance of EV-derived miR-122, it is essential to establish the biological context by explicitly delineating its changes in both hepatic tissue and total circulating compartments. As the first miRNA shown to directly regulate lipid metabolism, miR-122 is broadly involved in cholesterol synthesis and fatty acid metabolism and represents a key component of the hepatic energy metabolic network [44,45]. Within hepatocytes, miR-122 expression is controlled by multiple transcription factors and signaling pathways, including retinoic acid receptor-related orphan receptor a (RORA) and CCAAT/enhancer-binding protein α (C/EBPα), as well as pro-fibrotic signals such as transforming growth factor-beta (TGF-β) and the long ncRNA nuclear enriched abundant transcript 1 (*NEAT1*). In addition, evidence suggests that miR-122 enhances insulin signaling by targeting protein tyrosine phosphatase 1B (PTP1B), thereby contributing to the maintenance of hepatic insulin sensitivity [46].

In patients with MASL/MASH, hepatic miR-122 is markedly downregulated. Compared with SS, miR-122 expression in NASH liver tissue is decreased by approximately tenfold [47]. This downregulation of miR-122 is closely associated with disrupted lipid metabolism. Mechanistically, miR-122 regulates lipid synthesis and metabolism by targeting sterol regulatory element-binding proteins (*SREBPs*) and 3-hydroxy-3-methylglutaryl-coenzyme A reductase (*HMGCR*). Hepatocyte-specific miR-122 knockout in mice leads to hepatic accumulation of cholesterol and triglycerides despite reduced serum lipid levels. This observation highlights an essential role for miR-122 in lipid transport processes, including very-low-density lipoprotein (VLDL) assembly and secretion [46]. Downregulation of miR-122 has been shown to increase the expression of lipogenic genes such as *FASN*, acetyl-CoA carboxylase (*ACC*), and stearoyl-CoA desaturase 1 (*SCD1*), thereby promoting lipid accumulation in hepatocytes [46].

In contrast to its downregulation in hepatic tissue, total circulating (serum) miR-122 is significantly elevated in patients with MASL/MASH. In NASH patients, miR-122 is elevated approximately 7.2-fold, distinguishing simple steatosis from NASH [47]. Circulating miR-122 levels rise with the progression of steatosis and inflammation, positively correlating with alanine aminotransferase (ALT), aspartate aminotransferase (AST), inflammatory scores, and NAFLD activity score (NAS) [48,49]. They also show significant correlation with the apoptosis marker cytokeratin-18 (CK-18), suggesting a close association with caspase-mediated hepatocyte apoptosis. Its diagnostic performance in differentiating NASH and predicting fibrosis is superior to CK-18 and slightly better than traditional liver enzymes. Circulating miR-122 levels are also significantly associated with hepatocellular ballooning, further supporting its functional involvement in NAFLD pathogenesis [47]. Experimental evidence shows that miR-122 inhibition can attenuate high-fat diet-induced inflammation and oxidative stress by targeting forkhead box transcription factor O3 (*FOXO3*) [50].

This opposing pattern (downregulation in liver tissue but upregulation in circulation) may reflect progressive hepatocellular injury, increased miRNA export, and disruption of intracellular homeostasis. It provides important context for understanding the active secretion and selective packaging of EV-derived miR-122 during disease progression.

Overall, miR-122 alterations in MASLD/MASH reflect both hepatocyte metabolic dysregulation and its role as an EV-encapsulated signaling molecule in inter-tissue communication. Compared with undifferentiated circulating miR-122, EV-derived miR-122 shows higher specificity and potential utility in disease staging and mechanistic studies. It could be used alone or in combination with other miRNAs to improve diagnostic accuracy [49].

### 5.2. MiR-1 Family

EVs released by steatotic hepatocytes are significantly increased in number, with a markedly altered miRNA cargo profile. Among these, the miR-1 family is selectively enriched in EVs, showing higher expression in EVs derived from steatotic hepatocytes than in those from control hepatocytes [51]. The miR-1 family primarily includes miR-1-1, miR-1-2, and miR-206. Although miR-1 and miR-206 originate from different genomic loci, they share the same seed sequence and are therefore often classified functionally within the same family.

Hepatocyte-derived EVs are particularly enriched in miR-1-3p, miR-1-5p, and miR-206. These EVs can be taken up by vascular endothelial cells, where overexpression of miR-1 mimics the pro-inflammatory effects of EVs, including upregulation of E-selectin, vascular cell adhesion molecule-1 (VCAM-1), and intercellular adhesion molecule-1 (ICAM-1) in response to TNF-α stimulation, increased monocyte adhesion, and activation of NF-κB signaling [51].

Mechanistically, EV-derived miR-1 directly targets and suppresses Kruppel-like factor 4 (KLF4), a key negative regulator of NF-κB transcriptional activity. Anti-miR-1 inhibitors can significantly reverse EV-mediated KLF4 downregulation and inflammatory activation, further confirming the critical role of the miR-1-KLF4-NF-κB axis in steatotic liver-associated vascular inflammation and atherosclerosis [51]. This mechanism also has pathological significance in vivo: in apolipoprotein E (ApoE)-deficient mice, administration of a miR-1-specific inhibitor (antagomiR-1) not only alleviated endothelial inflammation but also markedly reduced the formation of atherosclerotic plaques, suggesting that EV-derived miR-1 contributes to MASL/MASH-associated cardiovascular complications beyond in vitro inflammatory activation [51].

In summary, miR-1 family members enriched in hepatocyte-derived EVs play a key pro-inflammatory role in MASL/MASH-associated vascular inflammation and atherosclerosis by activating NF-κB via KLF4 suppression. Although miR-1 and miR-206 share a highly conserved seed sequence, they have distinct target gene profiles depending on tissue type and pathological context. Most studies to date have focused on miR-1, highlighting the need for further investigation of miR-206 and different miR-1 isoforms. Developing miR-1-targeted strategies, such as antagomiRs, holds promise for alleviating MASL/MASH-associated vascular inflammation and potentially preventing atherosclerosis [51]. However, this therapeutic approach has not yet been validated in human cohorts.

### 5.3. MiR-34a

MiR-34a is significantly upregulated during the progression of MASL to MASH. Its expression correlates with hepatic lipid accumulation, inflammation, and disease severity [52].

EV-derived miR-34a is closely associated with insulin resistance. In obese children with MASLD, EV-derived miR-34a-5p is significantly elevated and positively correlates with liver function markers (AST) and insulin resistance indices (triglyceride-glucose index (TyG), homeostatic model assessment for insulin resistance (HOMA-IR)), suggesting that it may serve not only as a disease biomarker but also as a reflection of hepatic metabolic and inflammatory status [53]. High-fat conditions upregulate miR-34a, suppressing enolase3 (*ENO3*) expression, impairing hepatocyte insulin signaling, and disrupting glucose metabolism, thereby promoting hepatic insulin resistance.

While direct evidence detailing the downstream targets of EV-derived miR-34a is still accumulating, exploring the regulatory networks of serum- and tissue-derived miR-34a provides an indispensable biological context. Hepatic tissue-derived miR-34a disrupts energy homeostasis primarily via the Sirtuin 1 (SIRT1)-adenosine monophosphate-activated protein kinase (AMPK)-Peroxisome proliferator-activated receptor α (PPARα) axis. It directly targets *SIRT1*, reducing its deacetylase activity and inhibiting AMPK activation, which decreases fatty acid oxidation and increases lipogenesis [54]. MiR-34a also downregulates PPARα and hepatocyte nuclear factor 4 alpha (HNF4α), impairing fatty acid transport and VLDL assembly, and limiting triglyceride export from the liver [55].

Overall, studies on miR-34a have confirmed that EV-derived miR-34a serves as a circulating metabolism-related biomarker. In contrast, its direct regulatory effects on hepatic lipid metabolism and energy homeostasis are mainly supported by studies conducted in liver tissue or total circulating miR-34a. Based on this, some studies have explored intervention strategies targeting miR-34a, such as a tetrahedral DNA nanostructure (TDN)-based miR-34a inhibitor delivery system, which enhances inhibitor stability and cellular uptake [56]. However, these approaches remain largely at the experimental stage, and their efficacy within an EV-specific context still requires further validation.

### 5.4. Others

Several additional EV-derived miRNAs have been shown to participate in hepatic lipid metabolism, inflammation, and immune regulation during MASL/MASH.

MiR-30a-3p is significantly enriched in small EVs derived from steatotic hepatocytes and suppresses ATP-binding cassette transporter A1 (*ABCA1*) expression by targeting the 3′UTR of *ABCA1*, reducing cholesterol efflux. This promotes macrophage foam cell formation and exacerbates atherosclerosis. In high-fat, high-cholesterol diet-fed *ApoE*^−/−^ mice, inhibition of miR-30a-3p markedly attenuates atherosclerosis progression [57]. Senescent cell-derived EVs are enriched in miR-30b-5p, which can activate the NF-κB pathway by inhibiting SIRT1 and thereby enhancing the production of pro-inflammatory cytokines. This provides a potential mechanistic link to aging-related hepatic inflammation [58].

MiR-181d-5p mediates hepatoprotective effects via the muscle-liver axis through EV transport. Overexpression of miR-181d-5p suppresses nuclear receptor subfamily 4 group A member 3 (NR4A3), improving steatohepatitis and hepatocyte transcriptome dysregulation. Circulating EVs from volunteers subjected to remote ischemic conditioning (RIC) similarly ameliorate fatty liver pathology in mice, highlighting the translational potential of EV-derived miR-181d-5p in human liver protection [59].

Furthermore, several EV-derived miRNAs, including miR-135a-3p, miR-129b-5p, and miR-504-3p, are significantly reduced in the serum of high-fat diet-fed mice. Notably, a similar expression pattern of miR-135a-3p has also been reported in patients with MASLD, supporting its potential value as an early disease marker [60].

MiR-192-5p has been reported to participate in hepatic lipid metabolic regulation through multiple pathways, including targeting *SCD1* to modulate lipid synthesis [61], regulating hepatic triglyceride metabolism via Yin Yang 1 (*Yy1*) [62], and improving hepatic steatosis through a glucocorticoid receptor-related pathway [63]. EVs-derived miR-192-5p from fatty hepatocytes has been shown to activate macrophages through the Rictor/Akt/FoxO1 signaling pathway, contributing to immune activation and metabolic imbalance [64,65].

At the MASL/MASH stage, it is noteworthy that several well-characterized miRNAs are associated with dysregulated lipid metabolism and enhanced inflammation. For instance, miR-21 functions through the phosphatase and tensin homolog (PTEN)/Akt signaling pathway. Downregulation of PTEN activates Akt signaling, which promotes the expression of lipogenic genes such as *SREBP-1c* and *FASN*, thereby exacerbating hepatic steatosis [66,67]. Regarding inflammation, miR-21 enhances hepatic inflammatory responses by modulating the NF-κB pathway and pro-inflammatory cytokines, including IL-6 and TNF-α [66,67]. Circulating miR-193a-5p is elevated in MASLD patients and correlates with disease progression. MiR-378d is involved in the regulation of fatty acid oxidation and mitochondrial function, and changes in its expression are associated with hepatic lipid accumulation and insulin resistance [68]. In mouse models, miR-155 deficiency alleviates hepatic steatosis [69]. In contrast, miR-29a has a protective role in MASH. It improves mitochondrial function and reduces lipid accumulation and oxidative stress. It also regulates the Hippo pathway to suppress inflammation and hepatocyte pyroptosis, alleviating liver inflammation and fibrosis [70]. These circulating miRNAs provide important biological context for the MASL/MASH stage. However, whether their effects are mediated through EVs requires further investigation.

Overall, multiple EV-derived miRNAs play critical roles in dysregulated lipid metabolism, hepatocyte injury, inflammation, and immune regulation in MASL/MASH. EVs carrying miR-122, the miR-1 family, miR-34a, as well as miR-30a-3p, miR-30b-5p, and miR-181d-5p, not only reflect intrahepatic metabolic and inflammatory states but also transmit signals via circulating EVs, contributing to disease progression and potential cardiovascular complications. Some EV-derived miRNAs, such as miR-122 and miR-181d-5p, show consistent regulatory trends in both animal models and human samples, highlighting their translational potential as early disease biomarkers or therapeutic targets. Several circulating miRNAs, such as miR-21, miR-193a-5p, miR-378d, miR-155, and miR-29a, have not yet been clearly shown to act through EV-mediated mechanisms. During the MASL/MASH stage, they may be involved in lipid metabolism, inflammation, and apoptosis; however, their association with EVs still requires further validation.

However, most current data are derived from mouse models or limited human cohorts. The cellular origins of EV miRNAs have not been fully standardized, and experimental conditions vary widely. The dynamic changes in miRNAs in serum versus EVs, as well as their stage-specific expression patterns, remain incompletely characterized. In addition, confounding factors such as diet, metabolic status, comorbidities, and medication use may influence EV miRNA expression, limiting the interpretation of results.

Although some key EV miRNA regulatory circuits have been identified, such as miR-122/FASN/SIRT1, miR-1/KLF4/NF-κB, and miR-34a/SIRT1/AMPK/PPARα, the precise molecular mechanisms and causal relationships across different MASL/MASH stages require further validation. Circulating miR-122 has been reported to outperform ALT and AST in distinguishing MASH, yet the clinical advantages of EV-derived miRNAs as biomarkers remain to be demonstrated. Nevertheless, the stability, accessibility, and disease-specific expression patterns of EV-derived miRNAs make them ideal candidates for early diagnosis, risk stratification, and therapeutic intervention. These features provide a solid foundation for developing precision medicine strategies aimed at improving outcomes in MASLD patients.

## 6. EV-Derived miRNAs in Fibrosis and Cirrhosis

Fibrosis is a key factor in the development of HCC in patients with MASLD. Persistent inflammatory states in the liver lead to lipid accumulation, oxidative stress, and mitochondrial dysfunction. In this process, activated HSCs are converted into myofibroblasts, secreting pre-fibrotic mediators including TGF-β1, platelet-derived growth factor (PDGF), and connective tissue growth factor (CTGF), and synthesizing a large amount of ECM proteins, especially collagen [11]. This process eventually destroys the normal liver architecture, leading to fibrosis and eventually cirrhosis. As MASL/MASH progresses to liver fibrosis and cirrhosis, the hepatic microenvironment undergoes profound changes, and intercellular communication is remodeled. Increasing evidence indicates that during the fibrotic stage, the release of EVs from hepatocytes, macrophages, and adipose tissue-derived cells is significantly elevated, and the miRNA cargo of these EVs exhibits dynamic changes. Unlike free circulating miRNAs, EV-derived miRNAs offer greater stability and cell-specific delivery, and can be selectively taken up by HSCs, hepatocytes, and hepatic macrophages, thereby modulating fibrogenic processes.

In this section, we focus on EV-derived miRNAs and their functional mechanisms during liver fibrosis and cirrhosis.

### 6.1. MiR-21

As a critical biological context for understanding EV-mediated fibrogenic crosstalk, the functional focus of hepatic tissue-derived miR-21 shifts from metabolic regulation toward HSC activation and ECM deposition.

Both clinical and experimental studies have shown that miR-21 is significantly upregulated in liver tissue from patients with fibrosis and cirrhosis, and its expression level correlates positively with fibrosis severity [71,72].

Hepatic tissue-derived miR-21 promotes HSC activation by suppressing sprouty RTK signaling antagonist 2 (*Spry2*) and SMAD family member 7 (*Smad7*), thereby activating the extracellular signal-related kinase (ERK) and TGF-β1/Smads signaling pathways [73,74]. MiR-21 also regulates autophagy and metabolism-related pathways, for instance, through the FoxO3a/autophagy-related 5 (ATG5) axis, affecting fibrosis marker expression and modulating HSC activation and ECM accumulation [74]. In MASH mouse models, miR-21 knockout enhances PPARα activation and mitochondrial function, while improving inflammation and fibrosis [67].

At the EV level, fibroblast-derived EVs are enriched in miR-21, along with miR-124a, miR-125b, miR-126, miR-130a, and miR-132. These miRNAs can be taken up by neighboring cells and increase the expression of collagen α1 and α-smooth muscle actin (αSMA), promoting ECM accumulation in tissues and driving fibrotic progression [59]. These findings suggest that EV-derived miR-21 may act as an amplifier within the fibrotic microenvironment, strengthening intercellular signaling among HSCs. Compared with its metabolic roles during the MASL/MASH stage, EV-derived miR-21 in fibrosis is more directly involved in intercellular communication and maintenance of HSC function.

Although EV-miR-21 plays a key role in the fibrotic microenvironment, miR-21 is broadly expressed across multiple tissues and regulates diverse systems, including tumorigenesis, cardiovascular, and immune functions. Its limited specificity may constrain its use as a single biomarker. Therefore, distinguishing the functional roles of EV-derived miR-21 from different cellular origins and understanding their changes during disease progression could support more precise research and therapeutic strategies.

### 6.2. MiR-29

During the progression of liver fibrosis and cirrhosis, the miR-29 family, particularly miR-29a, is recognized as a classic anti-fibrotic miRNA and is markedly downregulated in fibrotic liver tissues [75].

TGF-β and NF-κB signaling has been shown to suppress miR-29 expression in HSCs, thereby allowing enhanced production of ECM proteins, including collagen type I alpha 1 (COL1A1), contributing to fibrogenesis [76,77]. Mechanistically, miR-29 directly targets ECM-related genes such as *COL1A1* and platelet-derived growth factor C (*PDGFC*) to inhibit collagen and other ECM components [75]. It also modulates the PI3K/Akt pathway to induce HSC apoptosis and reduce ECM accumulation, demonstrating a negative regulatory role in hepatic fibrogenesis in experimental models [78]. In vivo studies showed that exogenous administration of miR-29a suppresses HSC activation, decreases collagen deposition, and promotes fibrosis regression, indicating its potential as an anti-fibrotic intervention [75].

At the level of EVs, miR-29 exhibits more complex dynamic features. Studies have shown that under high concentrations of FFA stimulation, cellular miR-29a expression is downregulated, whereas its expression in EVs is upregulated [79]. This observation suggests a potential miRNA redistribution mechanism: intracellular miR-29 reduction may weaken its anti-fibrotic effect, while EV-derived miR-29 release could participate in intercellular communication, exerting regulatory effects across different cell types. Thus, the expression pattern of EV-derived miR-29 is not unidirectional but is influenced by multiple factors, including disease severity, metabolic status, and cellular origin.

Although the anti-fibrotic mechanisms of circulating miR-29 have been well studied, the expression and functional role of EV-derived miR-29 remain largely unclear. Most current evidence comes from animal models induced by carbon tetrachloride or thioacetamide, and clinical data from MASLD patients are still limited. Further investigation is needed to clarify the dynamic expression and cell type-specific mechanisms of EV-derived miR-29 at different stages of fibrosis. Future studies should focus on identifying the source cells, loading mechanisms, and stage-specific changes in EV-derived miR-29 to explore its clinical potential.

### 6.3. MiR-122

Unlike its role in lipid metabolism and inflammation, EV-derived miR-122 shows antifibrotic effects during the fibrosis and cirrhosis stages.

EVs derived from adipose tissue mesenchymal stem cells (MSCs) are enriched in miR-122. In CCl_4_-induced liver injury models, EV-mediated transfer of miR-122 significantly attenuates fibrosis progression, indicating a potential anti-fibrotic role for EV-derived miR-122 [41]. Furthermore, experimental studies demonstrate that adipocyte-derived EV-derived miR-122 not only participates in metabolic regulation but also exerts functional effects in inflammation and fibrogenesis, with inhibition of EV-derived miR-122 aggravating fibrotic phenotypes [42].

Meanwhile, miR-122 expression in the liver progressively decreases during the course of fibrosis. This reduction is particularly pronounced in patients with advanced fibrosis and cirrhosis, and hepatic miR-122 levels are inversely correlated with histological fibrosis stage and liver stiffness measurements [80,81]. TGF-β1 stimulation markedly downregulates miR-122 expression in HSCs, whereas restoration of miR-122 suppresses the transcription and protein expression of *α-SMA*, fibronectin 1 (*FN1*), and *COL1A1* [82]. MiR-122 directly binds to the 3′-UTR of *FN1* and inhibits its translation, and it also downregulates serum response factor (*SRF*) expression, decreasing collagen-related gene transcription [82]. Reduced miR-122 expression promotes hepatic stellate cell activation and fibrogenesis by relieving repression of pro-fibrotic targets such as *FN1*, *SRF*, Krüppel-like factor 6 (*KLF6*), and the collagen-modifying enzyme prolyl 4-hydroxylase aubunit alpha 1 (*P4HA1*). In parallel, long ncRNA NEAT1 can suppress miR-122 expression and indirectly enhance TGF-β-driven fibrotic signaling [46]. Hepatocyte-specific miR-122 knockout models exhibit progressive hepatic inflammation, increased collagen accumulation, and aggravated fibrotic phenotypes [83]. Therapeutic studies in animal models further demonstrated that mesenchymal stem cells overexpressing miR-122 significantly attenuate collagen deposition and reduce the number of α-SMA-positive cells in carbon tetrachloride (CCl_4_)-induced liver fibrosis, an effect associated with EV-derived miR-122 transfer to recipient cells [84]. Moreover, increased B cell lymphoma 2 (BCL2) expression has been observed in miR-122-deficient fibrotic models, and pharmacological inhibition of BCL2 alleviates liver fibrosis in this context [85].

The decrease in miR-122 within the liver, together with its abnormal export via EVs, may both contribute to fibrosis progression. During fibrotic development, intrahepatic miR-122 levels decline, while hepatocytes actively package miR-122 into EVs for secretion, leading to elevated levels of circulating EV-miR-122. This dynamic balance reflects both the metabolic and injury status of hepatocytes and endows EV-miR-122 with intercellular signaling functions.

Overall, miR-122 exerts anti-fibrotic effects and is consistently downregulated during liver fibrosis and cirrhosis. However, most available data are derived from experimental models or heterogeneous cohorts, and clinical studies specifically addressing EV-derived miR-122 in MASLD-related fibrosis remain limited. Prospective investigations employing liver-specific EV isolation strategies are warranted to further clarify the diagnostic and therapeutic potential of EV-derived miR-122 in MASLD-associated fibrogenesis.

### 6.4. Others

In addition to the well-characterized miRNAs described above, increasing evidence indicates that additional EV-derived miRNAs contribute to MASLD-associated liver fibrosis and cirrhosis. These miRNAs primarily modulate HSC activation and fibrogenic signaling pathways via EV-mediated intercellular communication. Unlike intracellular miRNAs, EV-derived miRNAs emphasize the crosstalk among metabolically dysregulated hepatocytes, inflammatory cells, and HSCs, forming a regulatory network underlying MASLD fibrosis.

MiR-34a expression correlates positively with fibrosis severity [86]. It can be induced by lipotoxic stress and inflammatory signals, enhancing pro-fibrotic interactions between hepatocytes and HSCs [87]. Although direct evidence for EV-derived miR-34a elevation in fibrotic or cirrhotic liver is limited, small EVs (sEVs) derived from adipose tissue macrophages (ATM) in MASH patients are enriched in miR-34a. These sEVs promote HSC activation in vitro, upregulating *Acta2*, *Col1a1*, *Tgfb*, *Ctgf*, and *Timp1*, and exacerbate liver fibrosis in obese mouse models in vivo [88]. Therefore, EV-derived miR-34a likely acts as an amplifier of the inflammatory-lipotoxic microenvironment rather than a direct ECM regulator.

MiR-155, a prototypical inflammation-associated miRNA, may indirectly promote HSC activation and ECM accumulation by amplifying inflammatory cues. Adipose tissue-derived EV-derived miR-155 targets PPARγ and influences hepatocyte insulin sensitivity [89], while MASH- adipose tissue macrophages (ATM)-derived sEVs enriched in miR-155 induce HSC fibrogenic gene expression [88].

Among EV-mediated communication studies, miR-192 is frequently reported as a pro-fibrotic miRNA. Hepatocytes exposed to fatty acids can deliver miR-192 via EVs to HSCs, upregulating α-SMA and COL1A1 expression and promoting ECM accumulation [90]. Hepatocyte-derived EV-derived miR-192-5p also activates macrophages through the Rictor/Akt/FoxO1 pathway, enhancing pro-inflammatory cytokine production and further driving the inflammation-fibrosis axis [64,89]. In vitro transfection of miR-192 into HSCs similarly increases fibrotic marker expression [65], supporting its functional role in fibrosis. EV-derived miR-192-5p thus acts both directly on HSCs and indirectly via immune modulation, highlighting its potential as a noninvasive biomarker and therapeutic target for MASH-associated fibrosis.

EV-derived miR-128-3p also exerts pro-fibrotic effects via hepatocyte-HSC paracrine signaling. Lipotoxic hepatocyte-derived EVs carrying miR-128-3p suppress PPARγ signaling in HSCs, promoting their activation, proliferation, and migration. Loss of EV-derived miR-128-3p leads to PPARγ upregulation and decreased expression of fibrotic markers, indicating its role as a critical negative regulator of the PPARγ pathway [41,91]. Mechanistically, EV-derived miR-128-3p primarily acts at the metabolic-transcriptional regulation level, distinct from inflammation amplification, and directly influences HSC phenotypic transformation. Clinically, EV-derived miR-128-3p may reflect early fibrotic risk driven by lipotoxicity.

EV-derived miR-214 has been demonstrated to regulate HSC activation and associated fibrotic signaling pathways. HSCs can secrete EVs containing miR-214, which inhibit CTGF (also known as cellular communication network factor 2 (CCN2)) expression in recipient HSCs or hepatocytes. miR-214 expression is transcriptionally regulated by Twist1, with Twist1 upregulation increasing miR-214 levels. Through EV-mediated transfer from donor HSCs to neighboring HSCs or hepatocytes, miR-214 targets the 3′-UTR of *CCN2*, thereby suppressing HSC activation and downstream fibrogenic gene expression [92]. In activated HSCs, both Twist1 and miR-214 are downregulated in EVs, suggesting that the Twist1-miR-214-CCN2 axis may constitute an important negative feedback mechanism controlling HSC activation [93]. Overall, EV-derived miR-214 exerts anti-fibrotic effects through EV-dependent intercellular communication, while its downregulation in activated HSCs may diminish this suppression, favoring fibrosis progression.

Accumulating evidence indicates that, beyond the classic miRNAs, other EV-derived miRNAs in MASLD-associated fibrosis display functional stratification: miR-34a and miR-155 primarily amplify inflammation and lipotoxic microenvironment signals; miR-192 and miR-128-3p directly drive HSC phenotypic transformation; and miR-214 represents a negative feedback regulator. These miRNAs act in a coordinated or antagonistic manner, collectively contributing to disease progression.

Overall, EV-derived miRNAs in MASLD-associated fibrosis and cirrhosis function as part of interconnected regulatory circuits rather than as isolated effectors. In the pro-fibrotic axis, EV-derived miR-21 amplifies HSC activation via SPRY2/Smad7-mediated modulation of the TGF-β/Smad signaling pathway; EV-derived miR-192 and EV-derived miR-128-3p directly drive HSC phenotypic transformation through the inflammation-macrophage axis and PPARγ-mediated metabolic-transcriptional regulation, respectively; EV-derived miR-34a and EV-derived miR-155 act primarily as amplifiers of lipotoxicity and inflammatory microenvironments, exacerbating HSC activation and ECM deposition. In contrast, EV-derived miR-29 and EV-derived miR-122 form anti-fibrotic regulatory axes, limiting fibrosis progression by directly suppressing ECM structural genes or maintaining metabolic homeostasis, while EV-derived miR-214 exerts inhibitory effects on excessive HSC activation via the Twist-CCN2 negative feedback pathway. Under the coordinated regulation of EV-derived miRNAs, HSCs transition from a quiescent to an activated state, secrete extracellular matrix components, and produce collagen and fibrous structures, ultimately leading to hepatic fibrosis. Progressive fibrosis results in pseudolobule formation and, ultimately, cirrhosis.

Based on the above mechanisms, EV-derived miRNAs not only serve as biomarkers for fibrosis progression but also represent potential therapeutic targets. Strategies aimed at modulating hepatocyte EV secretion, altering their miRNA cargo, or interfering with EV-HSC interactions may offer novel avenues for MASLD intervention.

## 7. EV-Derived miRNAs in HCC

Compared with MASL/MASH and fibrosis stages, EV-derived miRNAs in HCC not only participate in the molecular regulation of tumor initiation and progression but also show potential value in clinical diagnosis, prognostic evaluation, and prediction of treatment response.

### 7.1. EV-Derived miRNAs as Biomarkers for HCC Diagnosis and Prognosis

MASLD-associated HCC has been considered to arise through a multistep process, involving MASL, MASH, fibrosis, and cirrhosis. However, approximately one-third of MASLD-associated HCC cases arise in the absence of cirrhosis, and this subset of non-cirrhotic MASLD-associated HCC is often diagnosed at a later stage with a larger tumor burden [94]. The pathogenesis of non-cirrhotic MASLD-related HCC remains largely unknown and is likely multifactorial, potentially involving insulin resistance, low-grade chronic systemic inflammation, dysregulated lipid metabolism, local metabolic stress, gut microbiota dysbiosis, immune dysfunction, and sex hormone-related factors [95]. In this context, identifying reliable non-invasive biomarkers has important clinical implications.

Studies have found that EV-derived miRNAs exhibit different expression patterns in HCC patients compared to patients with chronic hepatitis B (CHB) or cirrhosis. The expression of miR-18a, miR-221, miR-222, and miR-224 in EVs of HCC patients was significantly higher than that of CHB or cirrhosis patients, while the expression of miR-101, miR-106b, miR-122, and miR-195 was significantly lower than that of CHB patients [10]. These differential expressions suggest that EV-derived miRNAs can be used as sensitive and non-invasive molecular biomarkers, compensating for the limited sensitivity of traditional alpha-fetoprotein (AFP) in the early stage of HCC [96].

EV-derived miRNAs also show diagnostic potential in non-HBV and non- hepatitis C virus-related HCC (NBNC-HCC) populations. A study in NBNC-HCC and NAFLD cohorts found that miR-19-3p, miR-16-5p, miR-223-3p, miR-30d-5p, and miR-451a were significantly elevated in plasma EVs from HCC patients. Among them, EV-derived miR-19-3p demonstrated high sensitivity for detecting AFP-negative and early-stage HCC and was identified as an independent unfavorable prognostic factor for overall survival. This finding indicates that EV-derived miRNAs may have not only diagnostic value but also potential utility in prognostic stratification [97].

Additionally, EV-derived miR-665 [98], miR-125b [99], miR-21 [100], etc., were correlated with HCC stage, survival, and prognosis. Therefore, EV-derived miRNAs may serve not only as diagnostic biomarkers but also for risk prediction and patient stratification.

It should be noted that most current studies do not strictly stratify HCC by etiology, with the majority of research conducted in overall HCC populations. However, recent studies focusing on NBNC-HCC populations are gradually emerging. Although strict etiological classification of HCC poses clinical challenges, investigating the pathophysiological processes underlying MASLD-associated HCC is of significant clinical importance. Whether EV-derived miRNAs are MASLD-specific remains to be further validated.

### 7.2. Roles of EV-Derived miRNAs in Tumor Microenvironment Remodeling and HCC Progression

At the HCC stage, EV-derived miRNAs not only serve as molecular biomarkers but also mediate communication between tumor cells, stromal cells, and immune cells, contributing to tumor microenvironment (TME) remodeling, apoptosis, angiogenesis, metastasis, and immune evasion [8].

This functional switch from tumor-suppressive to tumor-promoting highlights the plasticity of stromal cells, with EV-derived miRNAs being key drivers of this transformation. Moreover, HCC cell-derived EVs carrying miR-21 can induce HSC-to-cancer-associated fibroblast (CAF) conversion, further supporting TME formation [98]. These findings indicate that interactions between tumor and stromal cells are highly dependent on EV-derived miRNA signaling.

EVs derived from different cells carry different miRNAs and exert different biological functions. For example, normal HSC-derived EVs containing miR-335-5p can suppress HCC cell proliferation and invasion by regulating genes such as *CDC42*, *CDK2*, *CSNK1G2*, *EIF2C2*, *EIF5*, *LIMaK1*, *NRG1*, *PLK2*, *RGS19*, *TCF3*, *THBS1*, *YBX1*, and *ZMYND8* [101]. While in the TME, HSCs are transformed into CAFs. CAFs-derived EVs show a marked reduction in miR-320. The ability of miR-320 to inhibit the proliferation and migration of HCC cells was inhibited, thereby promoting tumor progression [102]. This functional transition from tumor-suppressive to tumor-promoting emphasizes the plasticity of stromal cells, with EV-derived miRNA being key factors driving this transition. HCC cell-derived miR-21 can induce the conversion of HSCs into CAFs, further promoting TME formation [103]. This shows that the interaction between tumor cells and stromal cells is highly dependent on EV-derived miRNA regulation.

In HCC cells, such as human hepatocellular carcinoma G2 (HepG2) cells, EVs show upregulation of miR-181a, miR-205, and miR-1323, while miR-23a, miR-16-2, miR-373, miR-27a, and miR-532 are downregulated [104]. These changes contribute to ECM remodeling and immune cell recruitment, thereby creating a microenvironment favorable for tumor growth and metastasis.

EV-derived miR-155 can not only promote neovascularization but also amplify intercellular communication in TME, participate in immune escape, and promote tumor growth [105]. Increased expression of miR-155 can promote the release of EVs from macrophages and hepatocytes [106]. EV-derived miR-142 and miR-223, originating from tumor-associated macrophages (TAMs), could inhibit HCC cell proliferation by regulating cell cycle-related genes, such as stathmin 1 (*STMN1*) [107].

Overall, current evidence suggests that EV-derived miRNAs, depending on their cellular origin and microenvironmental conditions, mediate signaling between tumor cells, stromal cells, and immune cells, potentially exerting tumor-suppressive or tumor-promoting effects.

### 7.3. Roles of EV-Derived miRNAs in Therapy Response and Drug Resistance

EV-derived miRNAs are also critically involved in modulating therapeutic response and drug resistance in HCC [108].

Some studies have found that miRNAs are involved in tumor resistance mechanisms by regulating cell proliferation, epithelial–mesenchymal transition (EMT), and immune response processes. Adipose tissue-derived mesenchymal stem cell (AMSC)-derived EVs carry rich miR-122, targeting genes such as *CCNG1*, *ADAM10*, and *IGF1R* in HCC cells. This subsequently enhances sensitivity to chemotherapeutic drugs and sorafenib. Intratumoral injection of miR-122-rich EVs can significantly improve the antitumor efficacy of sorafenib in vivo HCC [109]. Unlike its metabolic regulatory role during the MASLD stages, in HCC, EV-derived miR-122 primarily functions as an antitumor and chemosensitizing signaling molecule, reflecting a stage-specific shift in function.

MiR-744 is downregulated in serum EVs and tissues of HCC patients, and is further reduced in EVs from sorafenib-resistant HCC cells. Paired Box 2 (PAX2), a direct functional target of miR-744, is upregulated in HCC tissues and plays a significant role in weakening chemotherapy outcomes. Treatment of HCC cells with miR-744-enriched EVs markedly increased sorafenib sensitivity and reduced PAX2 levels, suggesting that EV-derived miR-744 could serve as both a biomarker and a therapeutic strategy for HCC by overcoming sorafenib resistance [110]. Similarly, miR-199a-3p has shown potential in enhancing doxorubicin’s antitumor effects [111].

On the other hand, some miRNAs promote drug resistance. For instance, macrophage-derived EVs have been reported to transfer miR-200c-3p to HCC cells, leading to activation of the PI3K/Akt pathway and reduced sensitivity to sorafenib [112].

These findings highlight the dual role of EV-derived miRNAs in precision medicine, where they can both enhance therapy sensitivity and promote drug resistance.

### 7.4. Specificity and Research Limitations of EV-Derived miRNAs in MASLD-Associated HCC

MASLD-associated HCC is increasingly recognized as a distinct HCC subtype with unique epidemiological features, molecular backgrounds, and carcinogenic pathways compared with viral- or alcohol-related HCC [113]. Multiple miRNAs have been associated with tumor initiation and progression in MASLD-HCC. However, it should be emphasized that most of these studies were based on tumor tissue or total circulating miRNA, without specifically analyzing EV-enriched fractions. For instance, miR-33 is upregulated and promotes tumor growth [114]; elevated miR-21 enhances tumor cell proliferation and survival, and increased miR-34a expression is associated with enhanced invasiveness and poor prognosis; and downregulation of miR-122 correlates with tumor growth and malignant progression [115,116]. Nevertheless, the majority of existing studies do not strictly stratify HCC by etiology, which limits current understanding of MASLD-HCC-specific miRNA profiles.

A defining feature of MASLD-HCC is its frequent occurrence in the absence of cirrhosis, suggesting carcinogenic drivers distinct from the classical “hepatitis-cirrhosis-HCC” pathway. However, research specifically addressing miRNAs involved in non-cirrhotic MASLD-HCC remains scarce. Most studies combine cirrhotic and non-cirrhotic MASLD-HCC cases, leaving the key miRNAs driving non-cirrhotic HCC largely undefined [113]. Moreover, heterogeneity in MASLD diagnostic criteria, cirrhosis assessment, and sample size further complicates systematic comparisons across studies [115]. Future investigations should therefore adopt strict etiological stratification to separately characterize miRNA expression profiles and oncogenic functions in cirrhotic and non-cirrhotic MASLD-HCC, thereby providing a robust basis for precision stratification and targeted intervention.

In summary, EV-derived miRNAs serve as versatile regulators in MASLD-HCC, integrating diagnostic, prognostic, and therapeutic functions. Their expression and biological effects are highly context dependent, influenced by cellular origin, tumor stage, and the microenvironment. Systematic elucidation of EV-derived miRNA-mediated mechanisms across different stages of HCC, particularly in tumor-stromal and immune cell interactions, will be essential for translating these findings into clinical applications. EV-derived miRNAs represent a critical link between HCC biology, TME remodeling, immune regulation, and clinical management, and deeper insight into these molecules may facilitate the development of novel diagnostic tools and targeted therapies to ultimately improve patient outcomes.

## 8. Cross-Stage EV-miRNA Regulatory Circuits in MASLD to HCC Progression

Based on the discrete miRNA functions described above, the following summarizes their continuous, cross-stage regulatory networks during the MASLD-to-HCC progression. The progression from MASLD to HCC is not driven by isolated, stage-specific events, but rather by a dynamically evolving network of EV-derived miRNAs. Multiple key miRNAs are repeatedly detected across different disease stages, but their expression levels, cellular sources, and functional focus exhibit stage-dependent changes, forming a “cross-stage regulatory network”.

EV-derived miR-122 exemplifies the most prominent stage-dependent functional shift. During the MASL/MASH stage, EV-derived miR-122 primarily originates from hepatocytes and adipose tissue, where it regulates lipid metabolism genes and β-oxidation pathways to maintain intrahepatic lipid homeostasis and limit fat accumulation. Furthermore, its elevation in serum EVs also reflects the export of liver injury signals. As the disease progresses to fibrosis, intrahepatic miR-122 decreases, yet hepatocytes actively package miR-122 into EVs for secretion. Interestingly, EV-derived miR-122 from alternative sources, such as MSCs, demonstrates targeted anti-fibrotic properties by significantly attenuating fibrosis progression. In HCC, EV levels of miR-122 further decline, weakening its inhibitory effects on tumor proliferation, angiogenesis, and chemoresistance-related signaling, while also being associated with immune evasion and tumor microenvironment remodeling. Across the disease continuum, miR-122 appears to shift functionally from a metabolic regulator to an antifibrotic factor, and finally to a tumor suppressor.

While miR-21 primarily regulates metabolism and inflammation without clear EV-dependence in early stages. During fibrosis, fibroblast-derived EVs enriched in miR-21 are taken up by neighboring cells, activating HSCs by suppressing *SPRY2* and *Smad7*, thereby promoting ECM deposition and forming intercellular positive feedback signals. In the HCC stage, EV-derived miR-21 induces the conversion of normal HSCs into CAFs, thereby actively remodeling the TME to favor disease progression. The functional focus of EV-derived miR-21 appears to shift progressively from fibrosis toward the tumor axis, while its role within the metabolic-inflammatory axis remains to be fully elucidated.

Other molecules, such as EV-derived miR-192-5p and miR-34a, transition from acting as metabolic and immune stress amplifiers in MASLD to directly promoting HSC activation and structural fibrogenesis as lipotoxicity persists. These form multi-stage, intercellular regulatory chains.

Collectively, these cross-stage EV-derived miRNAs create a complex network that coordinates multiple targets and pathways, reflecting liver metabolism, inflammation, and fibrosis, while potentially providing pathological information or intervention targets at early HCC stages. Based on these features, cross-stage EV-derived miRNAs can serve as biomarkers and potential therapeutic targets for MASLD-to-HCC progression, offering a theoretical foundation for precision medicine strategies.

## 9. Challenges and Future Perspectives

**Figure 1:**
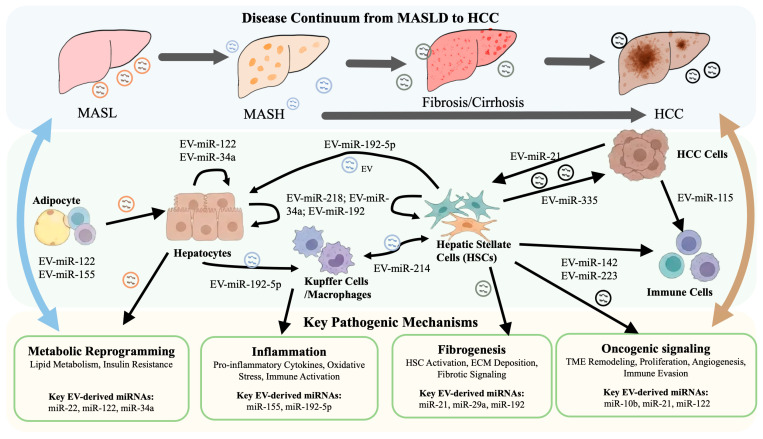
EV-derived miRNAs in the MASLD-to-HCC progression. This schematic illustrates the dynamic roles of extracellular vesicle (EV)-encapsulated miRNAs across the disease continuum, ranging from metabolic dysfunction-associated steatotic liver (MASL) to hepatocellular carcinoma (HCC). EVs act as critical mediators of intercellular communication, delivering specific miRNA cargos (e.g., miR-122, miR-34a, miR-192-5p, and miR-21) between hepatocytes, hepatic stellate cells, and immune cells. These EV-miRNA circuits orchestrate a complex network that drives lipid metabolic reprogramming, amplifies chronic inflammation, promotes fibrogenic remodeling, and reshapes the tumor microenvironment to favor malignancy. EV: extracellular vesicle; miRNA: microRNA; MASLD: Metabolic dysfunction-associated steatotic liver disease; MASL: metabolic dysfunction-associated steatosis liver; MASH: metabolic dysfunction-associated steatohepatitis; HCC: hepatocellular carcinoma; HSC: hepatic stellate cells; ECM: extracellular matrix; TME: tumor microenvironment.

MASLD represents a complex spectrum of metabolic liver diseases driven by lipid dysregulation, insulin resistance, inflammatory responses, and progressive fibrosis. Accumulating evidence indicates that miRNAs, as key post-transcriptional regulators, play critical roles throughout MASLD development and progression. Benefiting from the relative stability and detectability of EVs in circulation, EV-derived miRNAs serve as an important component of circulating miRNAs and mediate intercellular communication. They are involved in metabolic dysregulation, chronic inflammation, fibrotic remodeling, and TME remodeling (Figure 1). EV-derived miRNAs therefore represent promising biomarkers for early diagnosis and risk stratification and may also directly participate in disease-driving processes (Table 1). However, accurately distinguishing these functions from those of their tissue-resident or non-vesicular counterparts remains a significant challenge.

First, although EV-derived miRNAs are relatively stable and measurable in circulation, considerable heterogeneity exists across studies in EV isolation methods, miRNA detection platforms, and sample types (liver tissue, serum/plasma, or EV-enriched fractions). These methodological differences limit data comparability and reproducibility. Furthermore, EVs are biologically heterogeneous and originate from multiple cell types, including hepatocytes, adipocytes, macrophages, HSCs, and tumor cells. Their composition and function are strongly influenced by disease stage and pathological context, making it difficult to precisely identify key EV subpopulations and their miRNA cargo. While several representative EV-derived miRNA circuits are summarized in this review (Table 1), the complexity of the MASLD spectrum suggests that many critical pathways and phenotypic effects remain to be uncovered.

The progression from MASLD to HCC involves interconnected signaling pathways and regulatory layers, including inflammatory and immune signals, metabolic networks, and diverse intercellular interactions. This intrinsic complexity poses challenges for mechanistic studies. At present, most evidence is derived from in vitro experiments or animal models, with limited validation in large, well-characterized longitudinal clinical cohorts, thereby constraining translational potential.

Furthermore, most miRNA studies in HCC focus on “overall HCC” without stratification by etiological background, such as viral hepatitis, alcohol-related liver disease, or MASLD. MASLD-associated HCC exhibits distinct metabolic, inflammatory, and tumor microenvironment features, limiting the precision and generalizability of findings from non-specific cohorts. Notably, a substantial proportion of MASLD-associated HCC arises in non-cirrhotic livers, yet systematic studies of the independent mechanisms driving non-cirrhotic HCC and the regulatory networks of EV-derived miRNAs remain scarce.

MASLD is frequently accompanied by comorbidities such as type 2 diabetes, obesity, and cardiovascular disease. Although EV-derived miRNAs are relatively stable in circulation, standardized clinical evaluation is still lacking. Key issues include EV isolation protocols, miRNA quantification strategies, and control of confounding factors such as obesity, diabetes, medication use, and hemolysis. Large-scale longitudinal clinical studies are urgently needed to validate the diagnostic, staging, and prognostic value of EV-derived miRNAs and to establish standardized analytical workflows. Specifically, developing compartment-specific reporting standards that distinguish EV-encapsulated miRNAs from the total circulating pool will be crucial for clinical reliability.

Overall, EV-derived miRNAs show considerable promise as non-invasive biomarkers and therapeutic targets for MASLD and MASLD-associated HCC. Despite technical and mechanistic challenges, ongoing interdisciplinary research is expected to facilitate their translation from basic research to clinical application, providing new avenues for early intervention and precision management of MASLD and its related HCC.

## 10. Conclusions

Across the MASLD spectrum, EV-derived miRNAs exhibit marked stage-specific and dynamic expression patterns. In the MASL stage, alterations in EV-derived miRNA profiles primarily reflect the disruption of hepatic lipid homeostasis. During MASH, their roles expand to include the amplification of inflammation and the modulation of immune responses. In fibrosis and cirrhosis, EV-derived miRNAs drive HSC activation and extracellular matrix remodeling. At the hepatocellular carcinoma (HCC) stage, they participate in tumor microenvironment remodeling, activation of oncogenic signaling pathways, and promotion of tumor progression. Rather than acting as isolated molecular events, these miRNAs form a dynamically evolving regulatory network. The stage-dependent functional rewiring of key EV-miRNAs underscores the necessity of interpreting their expression patterns within specific pathological contexts. Furthermore, integrating findings across different biological compartments (from tissue-specific expression to EV-mediated secretion) is essential for a holistic understanding of the MASLD-to-HCC continuum.

Beyond serving as biomarkers of disease progression, EV-derived miRNAs represent potential targets for precision intervention. Although challenges remain in sample standardization, mechanistic clarification, and large-scale clinical validation, and etiology-specific or non-cirrhotic MASLD-HCC-focused studies are still limited, EV-derived miRNAs offer unique advantages for early diagnosis, risk stratification, disease staging, and therapeutic modulation. Future efforts integrating key EV-derived miRNA circuits, rigorous etiological stratification, and comprehensive preclinical and clinical validation will provide a solid foundation for precision diagnosis and treatment along the MASLD-HCC continuum.

## Figures and Tables

**Table 1:** Representative EV-derived miRNAs in MASLD-to-HCC Progression. MASLD: metabolic dysfunction-associated steatotic liver disease; HCC: hepatocellular carcinoma; MASH: metabolic dysfunction-associated steatohepatitis; HSC: hepatic stellate cells; ECM: extracellular matrix; MSC: mesenchymal stem cell; ATM: adipose tissue macrophages (↑: increased expression; ↓: decreased expression).

Disease State	miRNAs	Donor Cell Types	Modification	Major Targets/Pathways	Function	Biomarker Potential	Study Type	References
MASL	miR-122	Hepatocytes, ADEs	↑ (serum, EV), ↓ (hepatic)	SREBP, HMGCR, PTP1B	Regulation of lipid metabolism and liver energy homeostasis	Early diagnosis	Human, animal	[41,42,43,44,45,46,47,48,49,50]
	miR-1 family	Hepatocytes	↑	KLF4/NF-κB	Increased inflammatory signaling	Early inflammation	Cell, mouse	[51]
	miR-30a-3p	Hepatocytes	↑	ABCA1	Reduce cholesterol excretion and promote foam cell formation	Early markers	Cell, animal	[57]
MASH	miR-34a	Hepatic cell, ATM	↑	SIRT1-AMPK-PPARα, HNF4α	Metabolic imbalance, insulin resistance	Disease severity marker	Human, animal	[52,53,54,55,56]
	miR-192-5p	Hepatic cell	↑	*SCD1*, *Yy1*, FoxO1	Immune activation and lipid metabolism	Early markers	Cell, animal	[61,62,63,64,65]
	miR-181d-5p	Skeletal–liver axis	↑	NR4A3	Hepatoprotective effect	Translational potential	Animal	[59]
	miR-30b-5p	Senescent cell	↑	SIRT1/NF-κB	Inflammation amplified	-	Cell	[58]
Fibrosis/Cirrhosis	miR-21	Fibroblasts, HSC	↑	*SPRY2*/*Smad7*, ERK, TGF-β/Smad	HSC activation and ECM deposition	Progression, prognosis	Cell, animal, human	[59,71,72,73,74]
	miR-29a	HSC	↓ (hepatic), ↑(EV)	*COL1A1*, *PDGFC*, PI3K/Akt	HSC activation and ECM deposition	Intervention, flagging	Cell, animal	[75,76,77,78,79]
	miR-122	MSC	↑ (EV), ↓ (hepatic)	*FN1*, *SRF*, *COL1A1*, BCL2	Anti-fibrosis	Therapeutic potential	Cell, animal	[41,80,81,82,83,84,85]
	miR-128-3p	Hepatocytes	↑	PPARγ	HSC activation, proliferation, and migration	Early fiber risk	Cell, animal	[41,91]
	miR-214	HSC	↓	*CCN2*	Negatively regulates and HSC activation	Anti-fiber potential	Cell, animal	[92,93]
HCC	miR-221/miR-222/miR-224	-	↑	-	Diagnosis	Diagnosis, prognosis	Clinical	[10]
	miR-19-3p	-	↑	-	Early diagnosis and poor prognosis	Diagnosis, prognosis	Clinical	[97]
	miR-155	Tumor cells, Macrophages	↑	Immune and angiogenic pathways	Tumor progression	Prognosis	Cell	[105,106]
	miR-744	HCC cells	↓ (drug-resistant)	PAX2	Reverse drug resistance and improve sensitivity	Diagnosis, treatment response	Cell, human	[110]
	miR-200c-3p	M2 macrophage	↑	PI3K/Akt	Promotes primary resistance	-	Cell	[113]

## Data Availability

No new data were created or analyzed in this study.
